# Circadian Rhythm and the Seasonal Variation in Childhood Febrile Seizure

**Published:** 2017

**Authors:** Reza SHARAFI, Afagh HASSANZADEH RAD, Vahid AMINZADEH

**Affiliations:** Pediatrics Growth Disorders Research Center, School of Medicine, 17th Shahrivar Hospital, Guilan University of Medical Sciences, Rasht, Iran

**Keywords:** Temperature, Circadian rhythm, Children, Febrile convulsion

## Abstract

**Objective:**

We aimed to assess the circadian rhythm and the seasonal variation in childhood febrile seizure (FS).

**Materials & Methods:**

This descriptive cross-sectional study was conducted retrospectively on patients’ records. Investigators assessed the records of patients with simple FS aged 6 to 60 months referred to Emergency Department of 17-Shahrivar Hospital, Rasht northern Iran during Jan 2010 to Jan 2013. Data were gathered by a checklist including age, sex, temperature, duration of seizure, seasonal, months, diurnal variation, and level of consciousness.

**Results:**

Totally, 349 patients including 193 (55.3%) boys and 156 (44.7%) girls with the mean age of 22.85±18.34 months were enrolled in this study. The mean temperature of patients was 38.45±0.53°C. The mean duration of seizure was 97.91±57 sec. Awake, drowsy and slept patients were noted in 170 (48.7%), 33(9.5%) and 146 (41.8%) cases, respectively. Most of the FS occurred in winter 118 (33.8%), afternoon 132 (37.8%) and in Jan 55 (15.8%).

**Conclusion:**

Body temperature adjusted by hypothalamus affecting by circadian rhythm. FS is the most common form of seizure in childhood occurred by multifactorial issues. Otherwise, the occurrence of seizure in patients with epilepsy may be affected by the circadian rhythm. Seizures happen more frequent at a specific time in 24 h during a day.

## Introduction

Febrile seizure (FS) is the most common form of pediatric seizure, which occurs in 2%-5% of children. Eighteen months old is the estimated peak age for its occurrence (1). FS is defined as the seizure with febrile illness during 6-60 months of age, which does not happen because of central nervous system infection or metabolic disorders.

Children do not have any history of afebrile seizures (2) and body temperature should be at least 37.8 °C (3).

FS is classified into simple and complex types. Simple FS is determined by generalized seizure with less than 15 min duration and one seizure during febrile illness. Yet the cause of FS is unknown. Genetic factors can affect the occurrence of this age-dependent seizure. Commonly most of the patients report the familial history of febrile seizure.

Usually, in most patients with positive familial history, FS happens as an autosomal dominant disease (2).

Otherwise, core body temperature has circadian rhythm during 24 h which varies from 36.5 °C (early morning) to 37.5 °C (early evening) (4). Specific cells in hypothalamus control the body temperature that varies by circadian rhythm. This rhythm emerges from at least 1 months of age in children (5).

From 18 months of age, the duration of morning nap gradually decreases and 6 yr old child has only nocturnal sleep (6), the circadian rhythm works properly during the specific age of FS (6 to 60 months) (5). Epileptic seizures occur mostly in some specific time of the day(7).

The seizures occurrence depends on circadian rhythm.

Circadian rhythm and the secondary cyclic changes in hormones and the sleep-wake cycles can have potential effect on the time of seizures occurrence (8).

Regarding the clinical experiences, the increased rate of seizure in patients with epilepsy may be associated with the nocturnal melatonin rise. Although the pharmacological level of melatonin prohibits the occurrence of seizure, the physiologic level of this hormone can induce seizure by inhibiting effect on dopaminergic system (inhibit seizure) (9). Furthermore, FS can be affected by seasonal variation (10-13).

In this study, we aimed to assess the circadian rhythm and the seasonal variation in childhood febrile seizure.

## Materials & Methods

This descriptive cross-sectional study was conducted retrospectively on patients’ records. The records of patients with simple FS aged 6 to 60 months referred to Emergency Department of 17-Shahrivar Hospital, Rasht, northern Iran during Jan 2010 to Jan 2013 were assessed.

Ethical approval was obtained from Guilan University of Medical Sciences (93072206, 15 October 2014) and consent letter was taken from all participants.

Data were gathered by a checklist including age, sex, temperature, duration of seizure, seasonal, months, diurnal variation, and level of consciousness. The axillary temperature was taken immediately or up to 30 min after FS and the temperature, more than 37.8 °C indicates fever.

The diurnal variation was mentioned by 4 classifications: morning (6 AM to 11:59 AM), afternoon (12:00 AM to 5:59 p.m.) evening (6:00 p.m. to 11:59 p.m.) and night (12:00 to 5:59 a.m.)

Wake-sleep pattern was noted as awake, drowsy and slept. Data were analyzed by descriptive statistics (number, percent, mean, standard deviation) in SPSS ver. 19 (Chicago, IL, USA).

## Results

Totally, 349 patients including 193 (55.3%) boys and 156 (44.7%) girls with the mean age of 22.85±18.34 were enrolled. The mean temperature of patients was 38.45±0.53 °C. Mean duration of seizures were 97.91±57 sec. Awake, drowsy and slept patients were noted in 170 (48.7%), 33 (9.5%) and 146 (41.8%) cases, respectively.

Most of the FS occurred in winter 118 (33.8%), evening 132 (37.8%) and in Jan 55 (15.8%) (Table 1).

**Table 1:** Seasonal, diurnal, and months variation of FS

**Seasonal variation**	**N (%)**
Spring	60(17.2)
Summer	93(26.6)
Autumn	78(22.3)
Winter	118(33.8)
Diurnal variation	
Morning	18(5.2)
Afternoon	123(35.2)
Evening	132(37.8)
Night	76(21.8)
Months variation	
January	55(15.8)
February	35(10)
March	28(8)
April	17(4.9)
May	20(5.7)
June	23(6.6)
July	34(9.7)
August	38(10.9)
September	21(6)
October	22(6.3)
November	14(4)
December	42(12)

## Discussion

Body temperature adjusted by hypothalamus affecting by circadian rhythm FS is the most common form of seizure in childhood occurred by multifactorial issues. 

Otherwise, the occurrence of seizure in patients with epilepsy may be affected by the circadian rhythm. 

Seizures happen more frequent at a specific time in 24 h during a day.

In this study, most of the seizures occurred at evening (6-12 p.m.) and were approximately 7 times greater than the occurrence of FS at morning. The result was consistent with the study that noted evening as the most frequent time for FS and it was 5 times higher than morning (10). Furthermore, Manferedini et al and Mikkonen et al mentioned the highest frequency of FS was mentioned at 6-12 p.m. (11, 13). This result may be noted regarding the normal circadian rhythm of body temperature. As the highest temperature commonly occurs at evening and afternoon, respectively, and this temperature may be near the mentioned threshold for FS (38.5C), it can lead to higher FS rate. However, panahandeh et al mentioned the highest frequency of FS was at afternoon and evening (12).

Results showed the lowest and highest frequency of FS at morning and evening. Comparing the mean duration of FS during morning and evening indicated no significant difference (morning 89.55±50.32 vs. evening 100.14±58.85 second) (P=0.470). Furthermore, there was no significant difference between morning and evening regarding the mean temperature (Morning 38.33±0.45 vs. evening 38.43±0.54 c) (P=0.470).

Therefore, circadian rhythm cannot change the seizure propensity. The mentioned result was similar with the previous study by Ogihara et al. The mean duration of FS at morning and evening was 3.82 min and 3.14 min, respectively and the mean body temperature was 39.20 °C and 39.22 °C at evening and morning (10).

In this study, the highest frequency of FS occurred at winter and in January. This was similar to other results (11-13). This higher frequency may be because of higher frequency of febrile illness and infections during winter.

Most of the patients were awake during FS. Nevertheless, there were no previous investigations assessing the level of consciousness in patients. 

The incidence of FS was higher in specific seasons and months (especially during the winter), certain times of the day, and certain sleep-awake states. As daily and long-term anticonvulsant therapy is not allowed due to its complications in childhood because could not prevent the occurrence of epilepsy, the best way to prevent febrile seizures is to administer diazepam at the time of the febrile illness.

**In Conclusion, **parents should be advised on taking more care of febrile illness in certain months, seasons or at certain times of the day and in certain sleep-wake states. 

They should prevent febrile seizures by prophylactic administration of diazepam (due to short-term effect of diazepam).
